# Ultra-low reflectance, high absorption microcrystalline silicon nanostalagmite

**DOI:** 10.1186/1556-276X-7-171

**Published:** 2012-03-06

**Authors:** Subramani Thiyagu, Balasubramaniam Parvathy Devi, Zingway Pei, Yu-Hung Chen, Jun-Chin Liu

**Affiliations:** 1 Graduate Institute of Optoelectronic Engineering, Department of Electrical Engineering, National Chung Hsing University, Taichung, Taiwan, 40227, Republic of China; 2 Photovoltaic Technology Division, Green Energy and Environment Research Laboratories, Industrial Technology Research Institute (ITRI), Hsinchu, Taiwan, 31040, Republic of China

**Keywords:** ultra-low reflection, microcrystalline silicon, nanostalagmite, polystyrene nanospheres, light trapping

## Abstract

In this work, microcrystalline silicon nanostalagmite [μc-SiNS] arrays have been successfully fabricated on glass by catalytic etching process through a template. The template, polystyrene [PS] nanospheres, with diameter and density of 30 to approximately 50 nm and 10^10^/cm^2^, respectively, was obtained by a modified nanophase separation of PS-containing block copolymer. The length of μc-SiNS could be controlled by the duration of etching time. The μc-SiNS exhibits ultra-low reflection approximately 0.3% and absorption around 99% over 300 to 800 nm in wavelength. Reflection is also suppressed for a wide range of angles of incidence in wide range of wavelength. This indicates the extensive light-trapping effect by the μc-SiNS and could possibly harvest a large amount of solar energy at infrared regime.

## Introduction

Silicon thin film solar cells are promising candidates for future generations of photovoltaic devices [1,2]. They offer cost effectiveness and the possibility of deposition on flexible substrates [3-5]. However, the efficiency of thin film Si solar cell is relatively low compared to crystalline solar cell. The low absorption rate, relative poor material quality and narrow absorption spectra are the major factors. In the past few years, there is an enormously growing interest in the development of nanostructure materials to improve the light-harvesting efficiency for achieving high-efficiency Si thin film solar cell while maintaining low cost. Feasible silicon nanostructures such as silicon nanowires [SiNWs] have gained much attention due to their unique properties and possible applications in the fields of nanoelectronics [6-9], nanooptoelectronics [9,10], nanophotovolatics [11-17] and for sensor applications [18]. SiNWs are usually produced via vapour-liquid-solid [VLS] growth mechanism [19], which introduced one-dimensionality growing of nanostructure by a metal nanocatalyst droplet containing gases such as silane or grow from the gas phase by supplying Si vapour. However, the VLS growth mechanism generally requests high temperature that is not the adequate method for Si thin film nanostructure. In particular, the microcrystalline silicon [μc-Si] solar cells grown on glass or plastic substrate cannot sustain high temperature.

Nanoelectronics and nanooptoelectronics require vertically oriented, length tuneable and high density silicon nanostructures to obtain processing compatibility. To obtain such nanostructure, since some of the VLS growth does not occur, the wet chemical etching [20-23] through a predetermined template might be a possible method to achieve light-trapping structure at low cost. An efficient light management is essential to further improve the light confinement in the cells. Light trapping is the standard technique for improving thin film silicon efficiencies and exploiting the sunlight. In particular, μc-Si solar cells have gained considerable attention in the recent years. Wet chemically etched μc-Si surfaces by catalytic etching method that forms the μc-Si nanostalagmite show an ultra-low reflectance compared to Si thin film layers. This ultra-low reflectance is potentially fascinating for photovoltaic applications where enough absorption of solar light occurs in thinnest Si layer possible. The μc-SiNSs structure can be used as solar cell absorber.

In this study, we show the wet chemical etching of silicon nanostalagmite arrays with controlled length, size and density with a potentially high throughput by 30 to approximately 50 nm polystyrene [PS] spheres in a convenient method. It exhibits a black appearance, and they are almost non-reflective due to strong light scattering and absorption inside the μc-SiNSs structure. The reflectance and transmittance of the μc-SiNS are about 0.3% in average over the spectral range of 300 to 800 nm.

### Experimental methods

**Figure 1:**
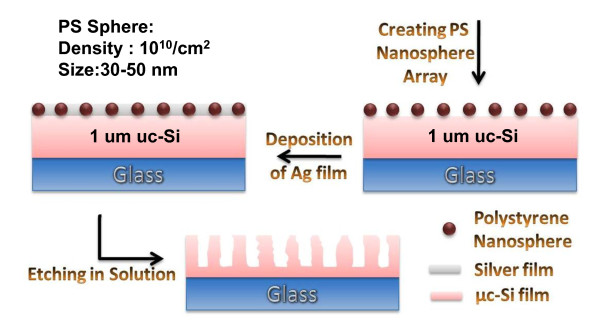
**Schematic diagram for the fabrication of microcrystalline silicon nanostalagmites**. Schematic diagram for the fabrication of microcrystalline silicon nanostalagmites using template polystyrene nanospheres based on modified block copolymer.

**Figure 2:**
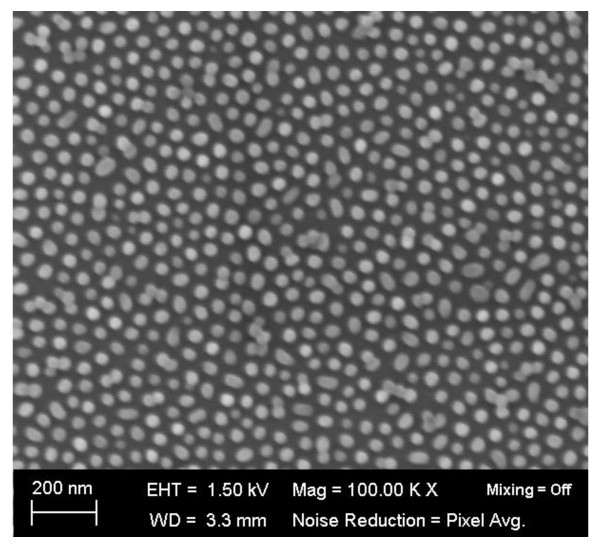
**SEM image of polystyrene nanospheres**. SEM image of polystyrene nanospheres with a diameter of 30 nm was used as a template to form nanostalagmites.

Figure 1 shows the schematic diagram for the fabrication of μc-SiNS arrays using a polystyrene nanospheres template obtained by the modified block copolymer nanopatterning method. SiNSs were prepared on indium-tin-oxide/glass substrate thorough a template by chemical etching with silver [Ag] catalyst. Initially, approximately 1 μm thick microcrystalline silicon [μc-Si] is deposited by plasma-enhanced chemical vapour deposition [PECVD] on glass substrate. The template used polystyrene nanospheres with a diameter of 30 to approximately 50 nm and density of 10^10^/cm^2 ^by nanophase separation of the PS-containing block copolymers as shown in the scanning electron microscope [SEM] image of Figure 2. The formation of PS nanospheres is implemented through a modified block copolymer (polystyrene-b- polymethylmethacrylate (acrylic) [PS-b-PMMA]) process by removing PMMA and reshaping the remained PS material [24]. After the formation of PS nanospheres on top of μc-Si, the silver film was deposited using thermal evaporator at the rate of 0.1 Å/s. The thickness of Ag film was 10 nm. A Teflon vessel was used as the container. For the solution etching process, an etching mixture consisting of hydrofluoric acid [HF], hydrogen peroxide [H_2_O_2_] and de-ionised water was used at room temperature. The concentrations of HF and H_2_O_2 _were 4.6 and 0.44 M, respectively. The Ag layers adhering to the Si surfaces had a higher electro-negativity than the Si, and electrons were therefore attracted to Ag from the Si, making the Ag layers negatively charged. The electrons from the negatively charged Ag layers were preferentially captured by the O^- ^ions of the H_2_O_2 _and become O^2- ^ions. This charge transfer caused local oxidation of the Si underneath the Ag patterns. The resultant SiO_2 _was then continuously etched away by HF, leading to the penetration of Ag into the Si substrates [16,25]. The μc-SiNSs are obtained when the Ag penetration reached a certain depth. The etching duration was varied depending on the required length of the nanostalagmites. After etching, the substrate was immersed in toluene for 1 h to remove the PS nanospheres. The silver film was then removed by immersing it in boiling aqua regia (3:1 (*v/v*) hydrochloric acid/nitric acid) for 15 min.

## Results and discussion

**Figure 3:**
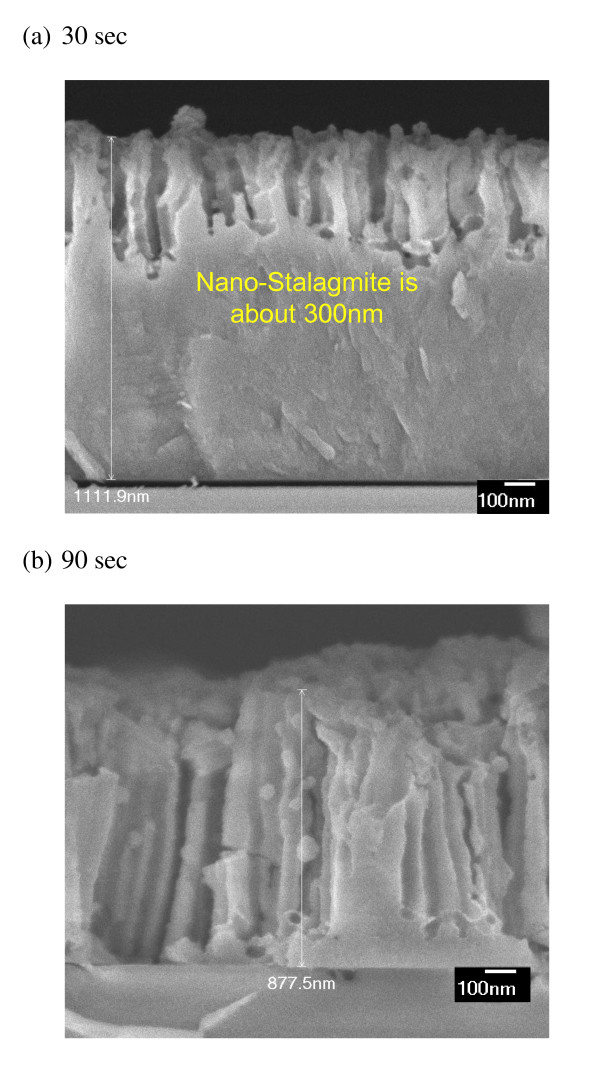
**SEM image of microcrystalline silicon nanostalagmite arrays**. High magnification cross-sectional SEM images of sample after etching for (**a**) 30 sec (**b**) 90 sec.

Figure 3 shows the SEM images of the silicon nanostalagmite array on the glass substrate. Length of SiNS can be controlled and varies linearly with the duration of catalyst etching process. Figure 3a, b shows high magnification cross-sectional SEM image of μc-SiNSs fabricated with different catalyst chemical etching times of 30 and 90 sec, producing nanostalagmites with a length of around 300 nm and 1 μm respectively. The SiNSs are randomly distributed due to PS nanosphere template which is aperiodic as shown in Figure 2. Although the SiNSs were not strictly vertical, this structure is still similar to that of the anti-reflection feathers on the eyes of moths [26,27].

**Figure 4:**
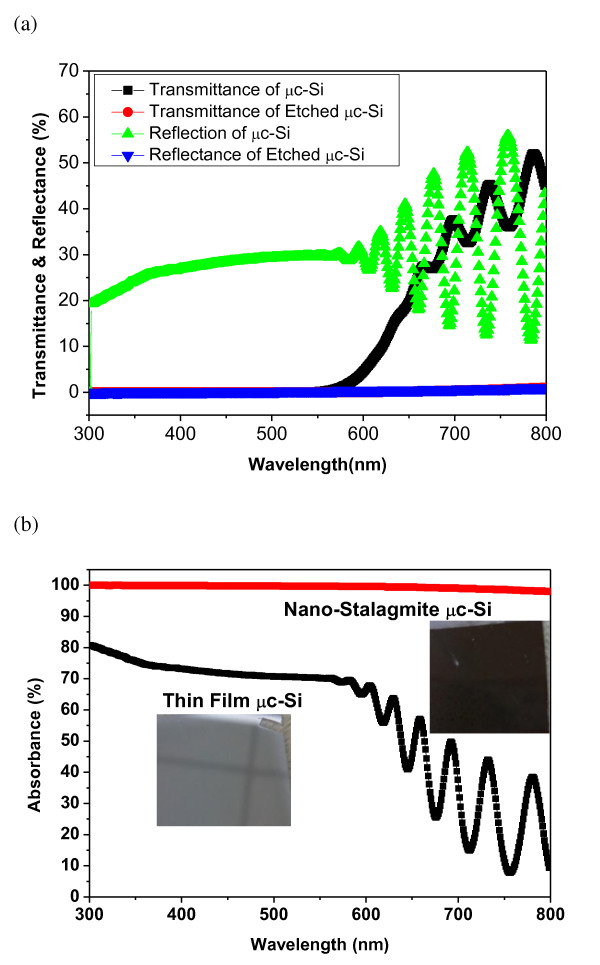
**Optical measurement on thin microcrystalline Silicon film with and without nanostalagmites arrays: (a) transmittance and reflectance and (b) absorption**.

Nanowires provide not only the advantage of more efficient charge transport over planar material but also present the potential for improved optical absorption characteristics [27,28]. Nanostalagmites are similar in structure with nanowires. The SiNSs were expected to have an efficient light-trapping effect. The incident light will have multiple internal reflections to cause an optical path for light absorption. Optical measurements were performed on the microcrystalline samples before and after μc-SiNS fabrication. The prepared μc-SiNSs samples were black in appearance and highly non-reflective to the naked eye. Transmittance and reflectance spectra of thin μc-Si film before (light-grey) and after etching to form nanostalagmites (black) were shown in Figure 4a. The transmittance for μc-SiNS over the entire spectral range from 300 to 800 nm is around 0.3%. In comparison, the planar control shows increased transmittance and strong interference patterns after 600 nm. The reflectance of μc-SiNSs and μc-Si film were also depicted in Figure 4a. The ultra-low reflectance of the μc-SiNSs array, which is around 0.3% over 300 to 800 nm, was also observed. In comparison, the planar μc-Si film exhibits around 20% to 30% reflectance over the measured spectrum. The ultra-low reflectance of SiNSs is caused by a strong absorption due to the strong light-trapping property of the dense SiNSs. The nanostalagmite simply acts as a very effective anti-reflection layer. This might be due to the 'moth-eye-like' biomimetic effect.

**Figure 5:**
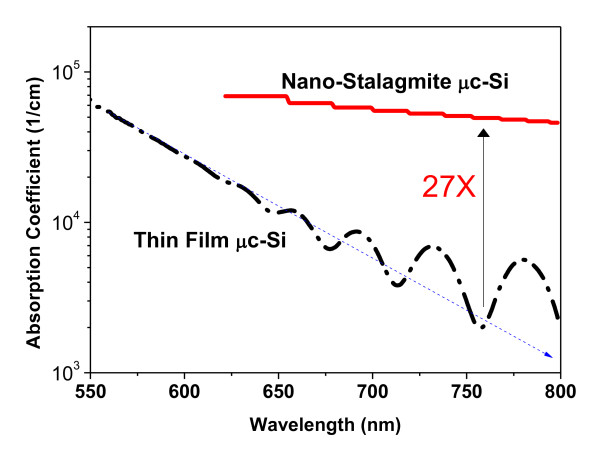
**The absorption coefficient for thin microcrystalline silicon film with and without nanostalagmites arrays**.

**Figure 6:**
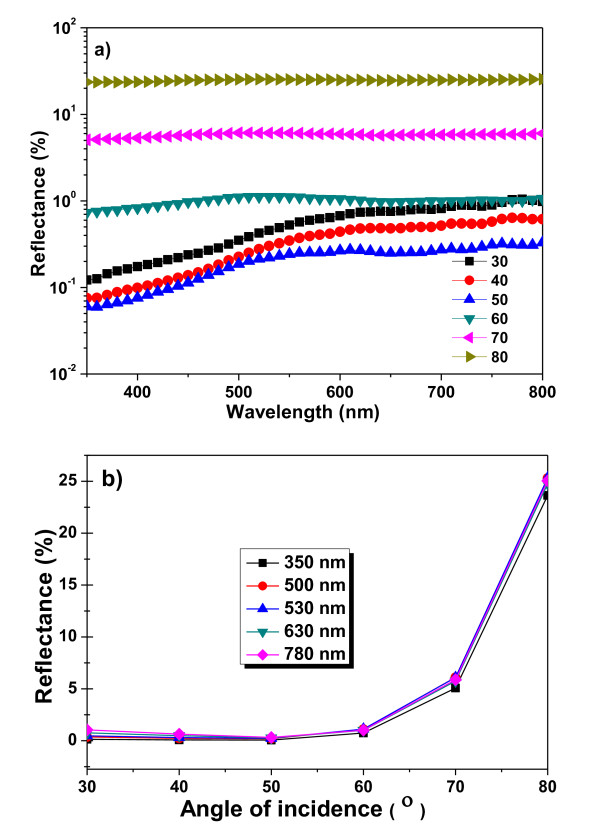
**Optical measurements of AOI and SiNS**. (**a**) Wide range of AOI in wide range wavelength (**b**) Angle-dependent reflectives of SiNS.

The acquisition of reflectance [*R*] and transmittance [*T*] spectra from μc-Si nanostalagmites allows further obtaining of their absorbance [*A*] spectra, expressed as *A*(%) = 100 - *R*(%) - *T*(%) as shown in Figure 4b. Photographs of bare μc-Si and a chemically etched substrate are shown in inset Figure 4b. The absorption over 98% was obtained for SiNSs over the measured spectrum from 300 to 800 nm. This indicates that the SiNSs could extend the absorption to the infrared regime, harvesting more light to increase photocurrent. Our nanostructure SiNS has low reflectance and high absorption; similarly, silicon nanocone [29] and nanodome [30] have higher absorption also. However, their process using reactive ion etching which means a high vacuum was used. Here, we demonstrated an alternative way by using simple and chemical process to get silicon nanostalagmite structure. This remarkable property suggests that SiNS arrays are appropriate candidates for antireflective surfaces and absorption materials used in photovoltaic cells. The effect of light trapping could be understood by the absorption coefficient. By taking the *R *and transmittance [*T*_0_] spectrum into account, the absorption coefficient can be calculated by the following equation:

(1) $$T=\frac{T_{0}}{\left(1-R\right)}=e^{-\alpha \cdot d}$$

in which *T *is the transmission, *α *is the absorption coefficient and *d *is the thickness of the μc-Si layer. Figure 5 depicts the absorption coefficient of SiNSs layer. The highest absorption coefficient for the SiNSs layer is also approximately 7 × 10^4^/cm at 620 nm. The absorption coefficient at wavelength shorter than 620 nm cannot be deduced because of the measured 'zero' transmittance, which is limited by the instrument. The absorption coefficient for the planar μc-Si is around 6 × 10^4^/cm at 550 nm. For the planar μc-Si, the absorption decreases with increase of the wavelength. However, after 600 nm, since planar μc-Si shows clear interference in the transmittance and reflectance spectra, the Equation 1 is not applied well. The absorption coefficient for μc-Si after 600 nm can only be used for estimation of the advantage of SiNSs layer. By this estimation, the average absorption coefficient for μc-Si at 750 nm is around 2 × 10^3^/cm. In comparison, the *α *value for SiNSs still retained around 6 × 10^4^/cm. There is about 27 times difference. This indicates that the light in the SiNSs at this wavelength is multiple-reflected 27 times by rough estimation. A good antireflective coating should show low reflectance over a wide angle of incidence [AOI], which is important for applications in sunrise-to-sunset solar cells. Figure 6a shows a wide range of AOI in the wide range wavelength. For all possible angles of incidence, we have measured in the range wavelength from 300 to 800 nm. One of the unique features of this catalytic chemical etching SiNS is that efficient light trapping occurs irrespective of the angle of incidence. Angle-dependent reflective of SiNS array was shown in Figure 6b. The performance of SiNSs arrays showed a reduced dependence on the angle of incidence and significantly higher absorption at any angle. At angle of incidence up to 60°, the total reflectance was maintained almost approximately 0.3%.

## Conclusions

In summary, we reported the fabrication of microcrystalline silicon nanostalagmite arrays on glass by catalytic etching process through 30 to approximately 50 nm PS nanosphere template. The length of nanostalagmite is defined by the duration of the etching process. The SiNSs arrays have low transmission, ultra-low reflection approximately 0.3% and high absorption around 99% compared to planar due to their strong light-trapping effect. Reflection is also suppressed for a wide range of angles of incidence in wide range of wavelength. This indicates the extensive light-trapping effect by the μc-SiNS and could possibly harvest a large amount of solar energy at infrared regime. The photocurrent could be largely enhanced with thin μc-Si layer in the future.

## Abbreviations

μc: microcrystalline; Ag: Silver; HF: hydrofluoric acid; H_2_O_2_: hydrogen peroxide: PECVD: plasma enhanced chemical vapour deposition; PS: polystyrene; SEM: scanning electron microscope; SiNS: silicon nanostalagmite.

## Competing interests

The authors declare that they have no competing interests.

## Authors' contributions

ST and ZP conceived of the study and participated in its design and coordination. ST and BPD carried out the experiments on fabrication and SEM/optical measurements of the nanostalagmites. YHC and JCL carried out PECVD experiments. ST drafted the manuscript. ZP revised the manuscript. All authors read and approved the final manuscript.
